# Substrate specificity and kinetic mechanism of 3β-hydroxy-Δ^5^-C_27_-steroid oxidoreductase

**DOI:** 10.1016/j.jbc.2024.107945

**Published:** 2024-11-04

**Authors:** Sarah M. Gardner, Austin Vogt, Trevor M. Penning, Ronen Marmorstein

**Affiliations:** 1 Graduate Group in Biochemistry and Molecular Biophysics, Perelman School of Medicine University of Pennsylvania, Philadelphia, Pennsylvania, USA; 2 Abramson Family Cancer Research Institute, University of Pennsylvania, Philadelphia Pennsylvania, USA; 3 Department of Biochemistry and Biophysics, University of Pennsylvania, Philadelphia, Pennsylvania, USA; 4 Department of Systems Pharmacology and Translational Therapeutics, University of Pennsylvania, Philadelphia, Pennsylvania, USA

**Keywords:** metabolism, oxidoreductase, enzyme mechanism, membrane protein, cholesterol metabolism, bile acid synthesis

## Abstract

Cholesterol is a key sterol whose homeostasis is primarily maintained through bile acid metabolism. Proper bile acid formation is vital for nutrient and fat-soluble vitamin absorption and emulsification of lipids. Synthesis of bile acids occurs through two main pathways, both of which rely on 3β-hydroxy-Δ^5^-C_27_-steroid oxidoreductase (HSD3B7) to begin epimerization of the 3β hydroxyl of cholesterol into its active 3α conformation. In this sequence, HSD3B7 catalyzes the dehydrogenation of the 3β-hydroxy group followed by isomerization of the Δ^5^-cholestene-3-one. These reactions are some of the many steps that transform cholesterol for either storage or secretion. HSD3B7 has distinct activity from other 3β-HSD family members leaving significant gaps in our understanding of its mode of catalysis and substrate specificity. In addition, the role of HSD3B7 in health and disease positions it as a metabolic vulnerability that could be harnessed as a therapeutic target. To this end, we evaluated the mechanism of HSD3B7 catalysis and reveal that HSD3B7 displays activity toward diverse 7α-hydroxylated oxysterols. HSD3B7 retains its catalytic efficiency toward these substrates, suggesting that its substrate binding pocket can withstand changes in polarity upon alterations to this hydrocarbon tail. Experiments aimed at determining substrate order are consistent with HSD3B7 catalyzing a sequential ordered bi–bi reaction mechanism with the binding of NAD^+^ followed by 7α-hydroxycholesterol to form a central complex. HSD3B7 bifunctional activity is dependent on membrane localization through a putative membrane–associated helix giving insight into potential regulation of enzyme activity. We found strong binding of the NADH product thought to activate the isomerization reaction. Homology models of HSD3B7 reveal a potential substrate pocket that allows for oxysterol binding, and mutagenesis was utilized to support this model. Together, these studies offer an understanding of substrate specificity and kinetic mechanism of HSD3B7, which can be exploited for future drug development.

Cholesterol is a key sterol that is an essential precursor of all steroid hormones and bile acids (1, 2, 3). Cholesterol biosynthesis is tightly regulated, and homeostasis is maintained through dynamic processes of export and esterification. One of the main fates of cholesterol is primary bile acid synthesis, which is the predominant mechanism for clearance of excess cholesterol (4, 5). There are two primary bile acid synthesis pathways, the classical pathway that occurs in the liver and accounts for over 90% of bile acid synthesis and the alternative pathways, which occur extrahepatically, and account for the rest of bile acid synthesis (2, 6). These extrahepatic pathways produce oxidized metabolites, which can be further processed into bile acids after transport to the liver where the majority of bile acid synthetic enzymes are uniformly expressed (2).

3β-Hydroxy-Δ^5^-C_27_-steroid oxidoreductase (HSD3B7) is responsible for two key steps required to convert the 3β-hydroxyl group of cholesterol to the canonical 3α-hydroxy group characteristic of all bile acids, which then act as feedback inhibitors of their own synthesis (7, 8). HSD3B7 is the only enzyme known to date that has 3β-hydroxysteroid (3β-HSD)/isomerase activity toward C_27_ 7α-oxysterols (9). In the classical pathway, HSD3B7 catalyzes the dehydrogenation and isomerization of 7α-hydroxycholesterol (7α-OHC) to 7α-hydroxycholest-4-en-3-one, and in the alternate pathway, it is active toward 7α-hydroxysteroids in which the side chain is oxidized (10), thus making it a key enzyme in bile acid synthesis. Hydroxylation of the side chain diversifies the oxysterol pool and is necessary for its eventual shortening and conjugation to taurine or glycine for secretion. As the primary component of bile, bile acids play critical roles in digestion where they are essential for fat-soluble vitamin absorption and regulation of lipid metabolism (4, 11). Therefore, any disruption in the functionality of bile acid synthetic enzymes can lead to severe consequences for bile acid production and result in disease.

Bile acid synthesis disorders (BASDs) are characterized by nutrient malabsorption and cholesterol toxicity with defects in HSD3B7 being the most prevalent inborn error in bile acid metabolism (12, 13). Genetic defects in HSD3B7 cause extreme deficiencies in the production of primary bile acids, cholic and chenodeoxycholic acids, and increases production of hepatotoxic oxysterols (14). Patients with deficiencies often develop congenital bile acid synthesis defects and cholestatic liver, caused by the impairment in production and release of bile acids across hepatic membranes (12). Prior studies showed that HSD3B7 knockout mice are unable to epimerize the 3β-hydroxyl group present on cholesterol, resulting in noncanonical bile acid production (7). It was found that these bile acids resulted in decreased solubilization of nutrients and downstream dysregulation of bile acid metabolic genes. Interestingly, these adverse effects can be treated with oral cholic acid supplementation, which is currently the most effective treatment for individuals with BASD (15, 16, 17, 18). Effectively, treatment of BASD with bile acids not only allows for bile flow but can also induce negative feedback inhibition of cholesterol 7α-hydroxylase (*CYP7A1*) to halt production of hepatotoxic 7α-OHC (16, 18, 19, 20).

In addition to the crucial role that it plays in BASDs, HSD3B7 has recently been found by our group to be a potential metabolic vulnerability in clear cell renal cell carcinoma (ccRCC) (21). ccRCC is characterized by excess lipid droplet accumulation and was found to upregulate HSD3B7 to maintain cellular homeostasis (21). Depletion of HSD3B7 left ccRCC tumors vulnerable to toxic oxysterol build up, and pharmacological inhibition of HSD3B7 with celastrol phenocopied this effect (21). These findings implicate HSD3B7 inhibition as a potential therapeutic target for ccRCC, although the celastrol inhibitor is known to have off-target effects (22, 23). Therefore, an understanding of enzyme mechanism and substrate specificity is key to developing specific inhibitors of HSD3B7 and for ultimately understanding its mechanistic role in health and disease. Previous work studying HSD3B7 was limited because of the necessity for isolation from rabbit and pig microsomes (24, 25, 26). While both studies confirmed activity toward 7α-hydroxylated oxysterols, detailed kinetics and mechanistic insights were lacking.

HSD3B7 belongs to the short-chain dehydrogenase/reductase family 11E (SDR11E) and shares a conserved Rossman fold critical for NAD(P)^+^ binding, but sequence conservation varies greatly resulting in large mechanistic diversity, further necessitating detailed mechanistic studies (27). In addition, HSD3B7 shows substantial functional differences to other closely related family members within the 3β-HSD subfamily. In steroid metabolism, 3β-HSD dehydrogenase type I and II (HSD3B1 and HSD3B2, respectively) are primarily active toward C_19_ and C_21_ steroids and have significant differences in substrate specificities to HSD3B7 (10, 28, 29, 30, 31). Overall, these functional differences leave significant gaps in our understanding of HSD3B7 mode of catalysis and substrate specificity and how it relates to the larger enzyme family.

Here, we examined the preference of human HSD3B7 for 7α-hydroxylated oxysterols through comprehensive kinetic experiments. Our investigation determined steady-state kinetic parameters for individual oxysterols and NAD^+^. In addition, we gained insights into the kinetic mechanism and the requirement for membrane association of HSD3B7, which were supported by structural predictions, docking, and mutagenesis. These studies can be used to contribute to the development of HSD3B7 inhibitors by identifying particular enzyme–substrate complexes that could be specifically inhibited.

## Results

### Kinetic characterization of HSD3B7 reveals comparable activity for 7α-hydroxy oxysterol substrates

**Figure 1:**
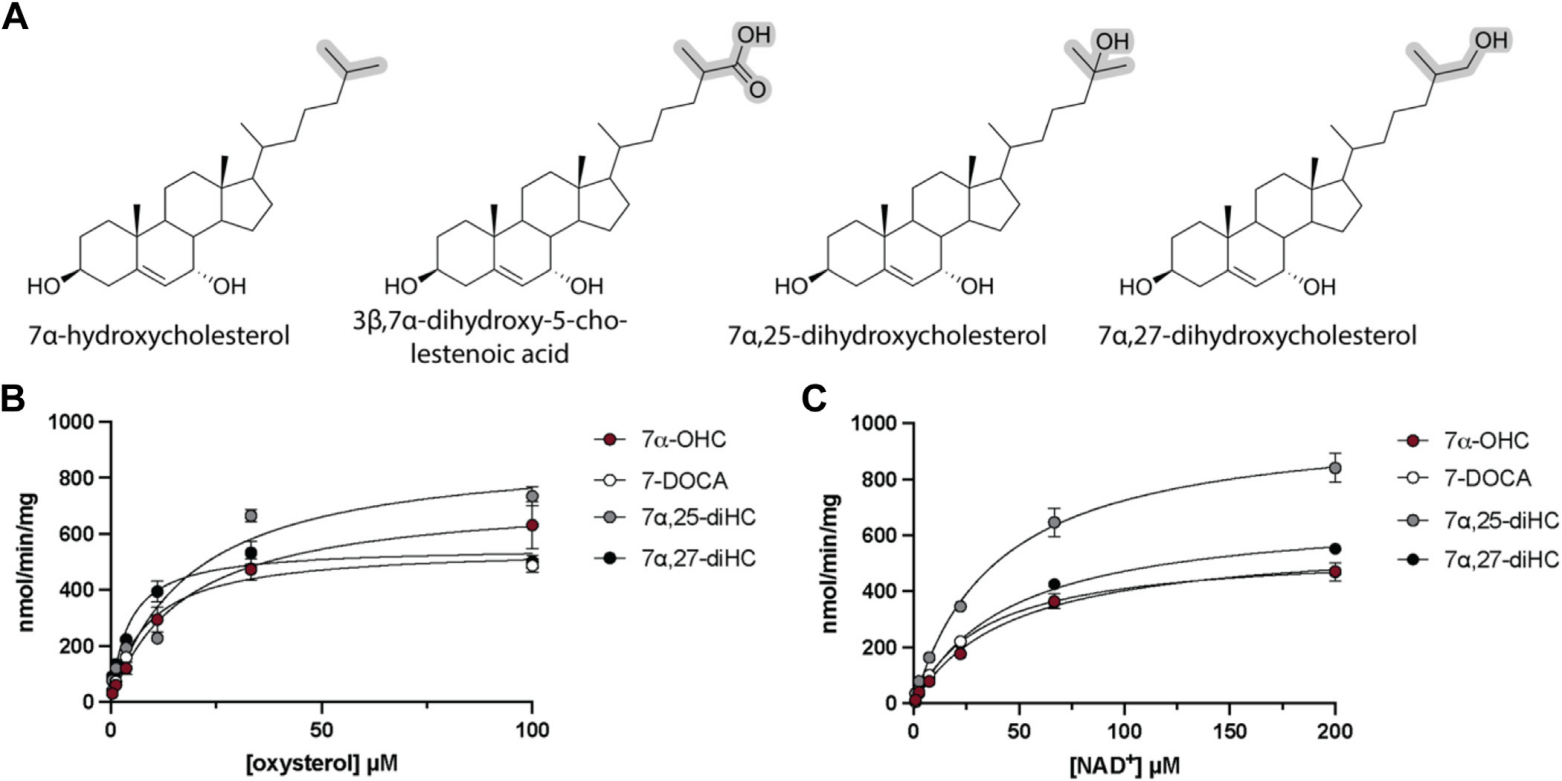
**Kinetic characterization of HSD3B7 for its 7α-oxysterol substrates and NAD+.***A*, structure of 7α-oxysterol substrates used in this study with varying modifications highlighted in *red*. *B*, steady-state enzyme kinetics for oxysterol substrates and HSD3B7 with constant NAD^+^. *C*, steady-state enzyme kinetics for NAD^+^ and HSD3B7 with constant oxysterol substrate. HSD3B7, 3β-hydroxy-Δ^5^-C_27_-steroid oxidoreductase.

Recombinant WT HSD3B7 was expressed from a pFAST vector with an N-terminal 6x histidine tag using Sf9 cells and was purified through a series of purification steps, including detergent solubilization from the membrane fraction, affinity chromatography, and size exclusion. Based on the current literature, HSD3B7 is suspected to be active toward multiple 7α-hydroxy oxysterols. To assess substrate preference, steady-state kinetics were used to evaluate kinetic constants for oxysterol and NAD^+^ cosubstrates. In addition to the canonical 7α-OHC, we measured the kinetic constants for three oxysterols with a C_27_ oxidized side chain, 7-DOCA, 7α,25-diHC, and 7α,27-diHC (Fig. 1*A*). The Michaelis–Menten equation was fit to the data for each oxysterol (Fig. 1*B*), and values are reported in Table 1. HSD3B7 is active toward multiple 7α-hydroxy bile acid precursors independent of whether their C_27_ tail is oxidized. Among the oxysterols evaluated, HSD3B7 has the lowest *K*_*m*_ for 7α,27-diHC over the classical pathway substrate 7α-OHC, which gave *K*_*m*_ values of 3.8 μM (95% confidence interval [2.8, 5.2]) and 17.4 μM [13.0, 23.4], respectively). Both 7-DOCA and 7α,25-diHC showed no significant differences in substrate *K*_*m*_ values relative to 7α-OHC. More importantly, we also found that the turnover number, *k*_cat_, remained consistent between oxysterols, suggesting that they share a common rate-determining step. The catalytic efficiency, *k*_cat_/*K*_*m*_, of HSD3B7 in the presence of 7α,27-diHC was 3.3-fold higher than that of 7α-OHC (Table 1). In contrast, 7-DOCA and 7α,25-diHC maintain similar catalytic efficiencies to that of HSD3B7 in the presence of 7α-OHC.

**Table 1:** Kinetic constants for oxysterols

Substrate	*K*_*m*_ (μM)	*V*_max_ (nmol/min/mg)	*k*_cat_ (min^−1^)	*k*_cat_/*K*_*m*_ (M^−1^ s^−1^) × 10^4^
7α-OHC	17.4 (13.0, 23.4)	735 (669.5, 812.3)	30.9 (28.1, 34.1)	3.0 ± 1.0
7-DOCA	8.6 (6.6, 11.0)	551.8 (513.5, 593.2)	23.2 (21.6, 24.9)	4.5 ± 1.7
7α,25-diHC	18.3 (12.0, 27.8)	901.8 (785.3, 1044)	37.9 (33.0, 43.9)	3.5 ± 1.2
7α,27-diHC	3.8 (2.8, 5.2)	532.0 (493.0, 573.4)	22.4 (20.7, 24.1)	9.8 ± 2.2

**Figure 2:**
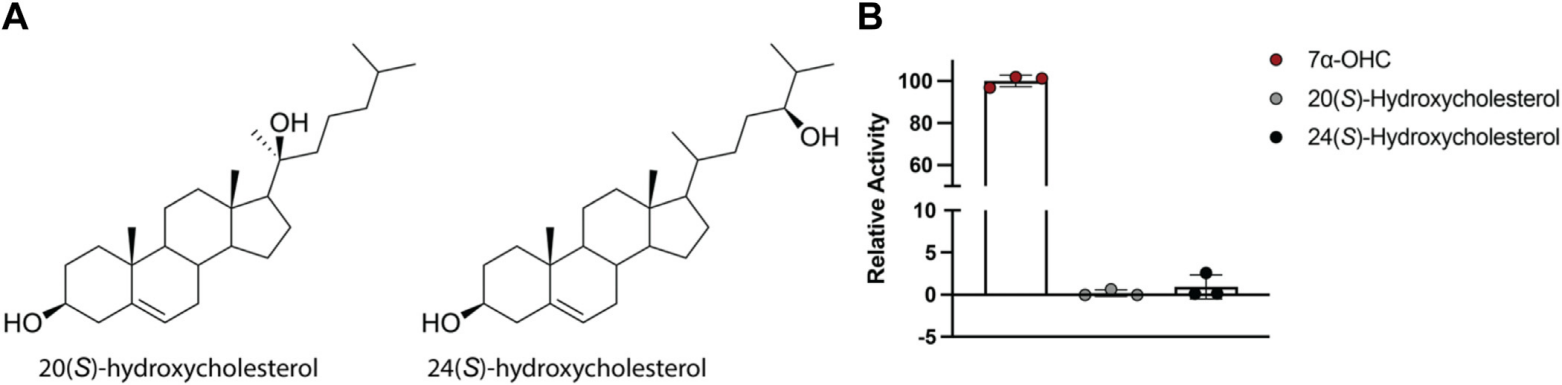
**7α-oxysterol specificity of HSD3B7.***A*, structures of 20(*S*) and 24(*S*)-hydroxycholesterol. *B*, activity assay of WT HSD3B7 in the presence of 7α-OHC, 20(*S*)-hydroxycholesterol, and 24(*S*)-hydroxycholesterol. The axis is adjusted to show low activity toward non-7α containing oxysterols. HSD3B7, 3β-hydroxy-Δ^5^-C_27_-steroid oxidoreductase.

Steady-state kinetics were also utilized to determine the kinetic parameters of HSD3B7 and NAD^+^ (Fig. 1*C*, Table 2). Differing oxysterol substrates do not alter the *K*_*m*_ of HSD3B7 for NAD^+^, resulting in values similar to that in the presence of the canonical substrate 7α-OHC (45.8 μM [38.1, 55.2]). HSD3B7 in the presence of excess 7α,25-diHC has a 1.7-fold increase in *k*_cat_ of 42.5 min^−1^ [39.9, 45.3] in comparison to 7α-OHC with a *k*_cat_ of 24.7 min^−1^ [23.1, 26.4], but the catalytic efficiency of HSD3B7 remains consistent, independent of oxysterol substrate (Table 2). Taken together, HSD3B7 shows comparable activity to 7α-hydroxy oxysterol substrates, as shown by similar catalytic efficiencies in the presence of these oxysterol substrates. To confirm the requirement of the 7α hydroxyl group for HSD3B7 activity, two non-7α hydroxy containing oxysterols were also evaluated for activity, 20(*S*)-hydroxycholesterol and 24(*S*)-hydroxycholesterol (Fig. 2*A*). As expected, neither oxysterol resulted in significant activity in comparison to 7α-OHC (Fig. 2*B*).

**Table 2:** Kinetic constants for NAD^+^

Substrate	*K*_*m*_ (μM)	*V*_max_ (nmol/min/mg)	*k*_cat_ (min^−1^)	*k*_cat_/*K*_*m*_ (M^−1^ s^−1^) × 10^4^
7α-OHC	45.8 (38.1, 55.2)	587.2 (550.0, 628.7)	24.7 (23.1, 26.4)	0.90 ± 0.32
7-DOCA	33.8 (29.0, 39.5)	545.8 (518.7, 575.0)	22.9 (21.8, 24.2)	1.1 ± 0.4
7α,25-diHC	39.5 (32.8, 47.5)	1010 (949.0, 1078)	42.5 (39.9, 45.3)	1.8 ± 0.6
7α,27-diHC	45.1 (39.9, 51.2)	684.8 (655.2, 716.7)	28.8 (27.5, 30.1)	1.1 ± 0.4

### HSD3B7 catalysis proceeds by a sequential ordered bi–bi mechanism with the binding of NAD^+^ followed by 7α-OHC

**Figure 3:**
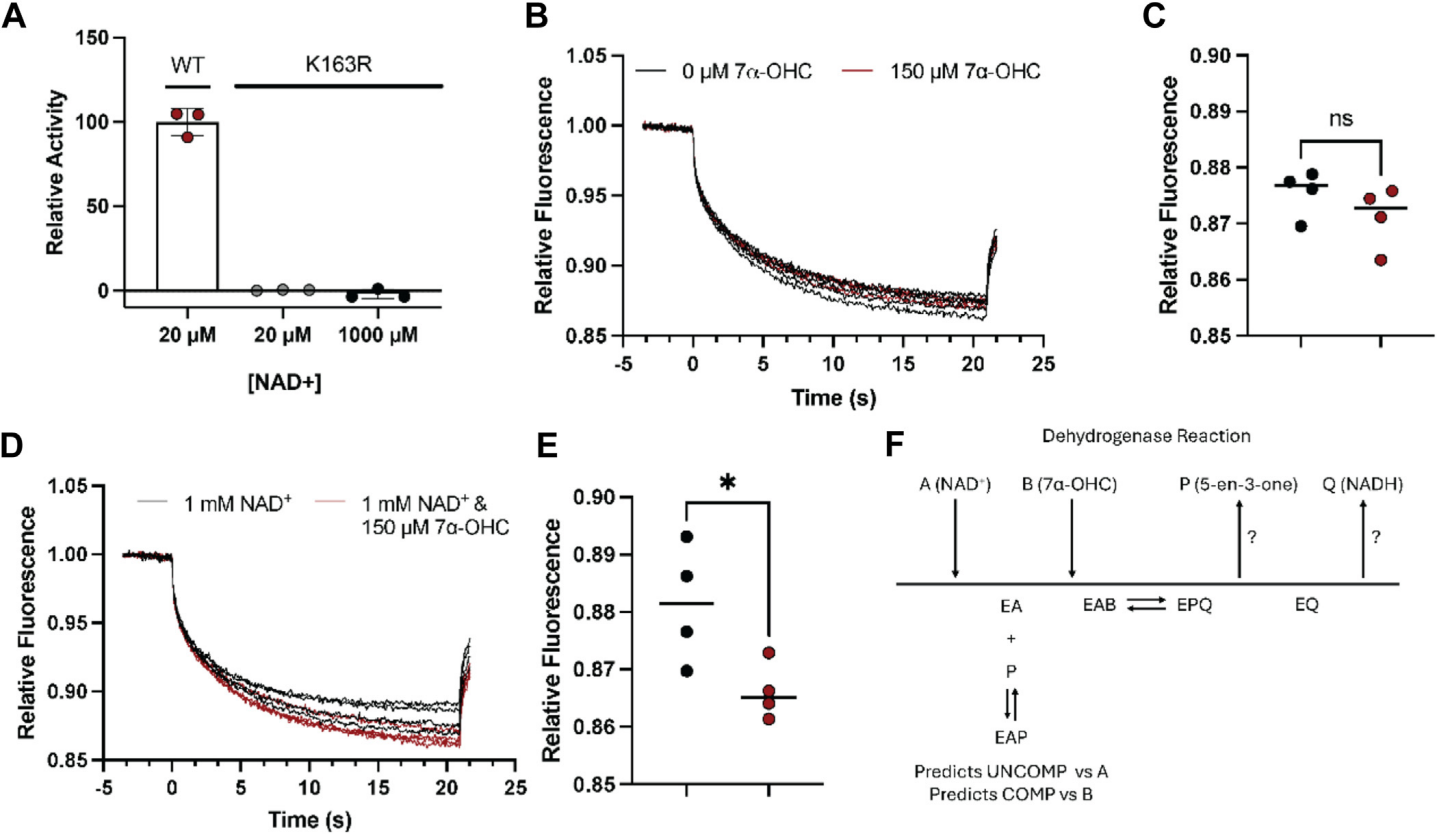
**Mechanism of HSD3B7 substrate binding.***A*, relative activity of catalytic mutant K163R. *B*, MST relative fluorescence traces of HSD3B7 in the presence (*red*) and absence (*black*) of 150 μM 7-HCO. *C*, MST relative fluorescence after 20 s in the presence (*red*) or absence (*black*) of 150 μM 7α-OHC. *D*, MST relative fluorescence traces of K163R with 1 mM NAD^+^ in the presence (*red*) and absence (*black*) of 150 μM 7α-OHC. *E*, MST relative fluorescence after 20 s of HSD3B7 with 1 mM NAD^+^ in the presence (*red*) or absence (*black*) of 150 μM 7-HCO. *F*, dehydrogenase scheme in Cleland representation. Order of product release was not evaluated. HSD3B7, 3β-hydroxy-Δ^5^-C_27_-steroid oxidoreductase; MST, microscale thermophoresis.

To evaluate the kinetic mechanism of HSD3B7, further biochemical experiments were warranted. Microscale thermophoresis (MST) was utilized to determine if HSD3B7 catalyzes an ordered or random mechanism, by evaluating shifts in migration in the presence and absence of NAD^+^ and substrate 7α-OHC using a catalytically inactive mutant K163R (Fig. 3*A*). Upon addition of 150 μM 7α-OHC, there were no significant changes in migration as seen by the raw MST traces, suggesting that 7α-OHC does not bind or binds weakly to unliganded HSD3B7 (Fig. 3, *B* and *C*). Upon incubation with 1 mM NAD^+^, a shift was seen in thermophoresis upon addition of 7α-OHC suggesting that NAD^+^ is required for 7α-OHC binding (Fig. 3, *D* and *E*). Together, these data support an ordered bi–bi substrate binding mechanism with the binding of NAD^+^ first and 7α-OHC second (Fig. 3*F*).

### NADH product binds tightly to HSD3B7

**Figure 4:**
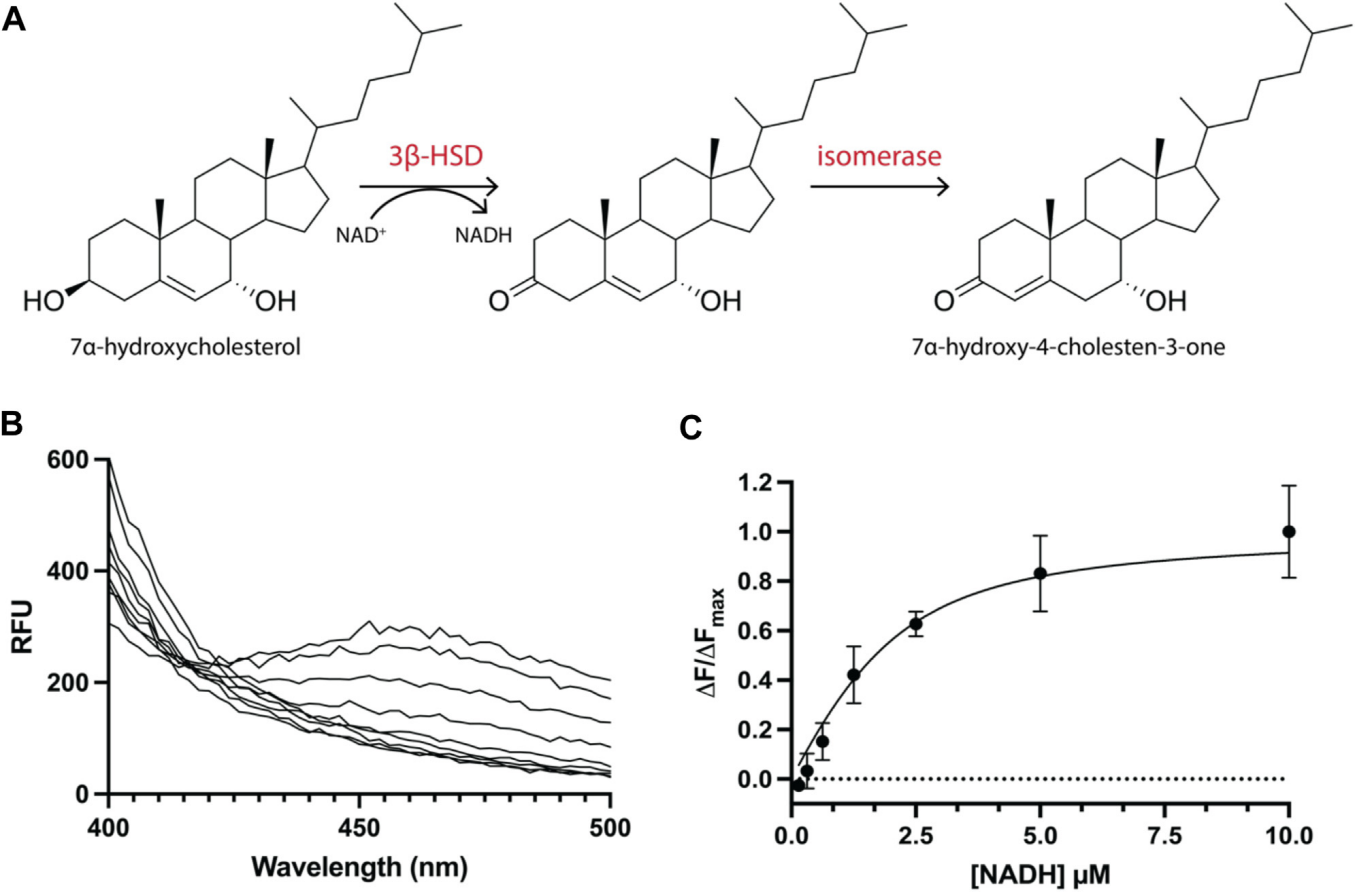
**Fluorescence emission spectra of HSD3B7 and titrated NADH.***A*, HSD3B7 reaction mechanism for 3β-HSD and isomerase activity. *B*, emission traces of varying NADH (0.15 μM–10 μM) in the presence of 1.8 μM HSD3B7 after excitation of the intrinsic tryptophan fluorescence. *C*, change in fluorescence at 460 nm *versus* NADH concentration fit to the Morrison equation. HSD3B7, 3β-hydroxy-Δ^5^-C_27_-steroid oxidoreductase.

HSD3B7 catalyzes a sequential reaction mechanism, with a dehydrogenase reaction preceding an isomerization reaction to produce the final products, NADH and 7α-hydroxycholest-4-en-3-one (Fig. 4*A*). We took advantage of properties of NADH and the intrinsic tryptophan fluorescence of HSD3B7 to determine the *K*_*d*_ of NADH for HSD3B7. The emission maximum of tryptophan overlaps with the absorption maxima of NADH at 340 nm, allowing for FRET to be measured. Upon excitation of HSD3B7 at 285 nm in the presence of NADH, there is a loss of fluorescence at 340 nm and an energy transfer band at 460 nm, because of bound NADH (Fig. 4*B*). After subtracting background fluorescence, the binding constant was fit using the Morrison equation (Equation 1a and Equation 1b) and resulted in a *K*_*d*_ of 0.78 μM [0.43, 1.3] (Fig. 4*C*).

### A predicted membrane-associated helix is essential for HSD3B7 catalysis

**Figure 5:**
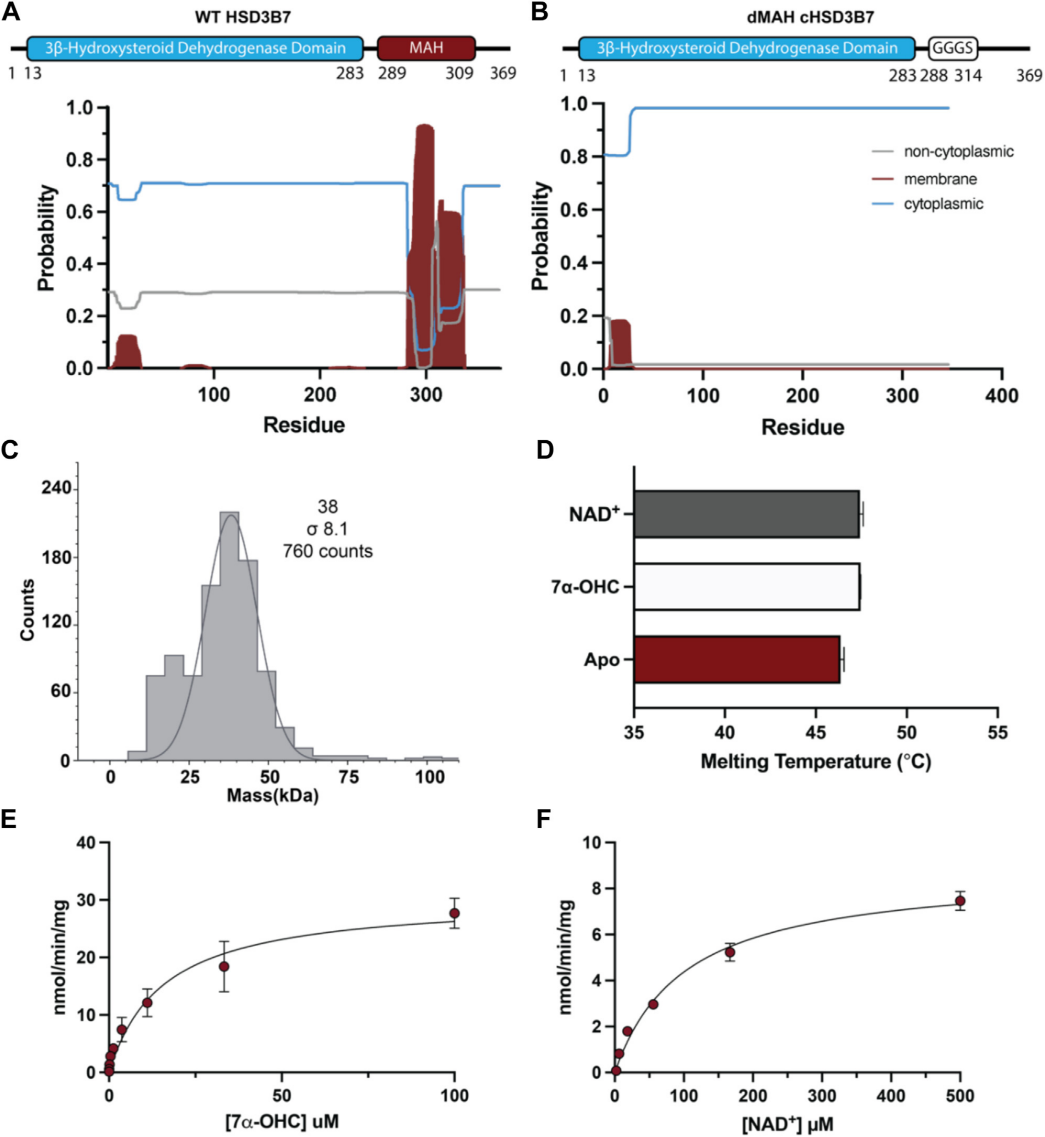
**Effect of membrane-associated helix (MAH) on HSD3B7 catalysis.***A*, domain architecture of WT HSD3B7 and 3β-HSD domain with deleted MAH and transmembrane helix hidden Markov model (TMHMM) for HSD3B7 sequence. Cytoplasmic, noncytoplasmic, and membrane-associated predicted sequences are represented by *blue*, *gray*, and *red*, respectively. *B*, second construct replaces the MAH with a GGGS linker and TMHMM for HSD3B7 sequence. Cytoplasmic, noncytoplasmic, and membrane-associated predicted sequences are represented by *black*, *gray*, and *red*, respectively. *C*, mass photometry of ΔMAH construct with a predicted molecular weight of 38 kD. *D*, thermostability of ΔMAH in the presence and absence of substrates. *T*_*m*_ of cHSD3B7-ΔMAH, cHSD3B7-ΔMAH + 7α-OHC, and cHSD3B7-ΔMAH + NAD^+^ is 46.4 ± 0.2, 47.5 ± 0.02, and 47.4 ± 0.2 °C, respectively. *E*, steady-state kinetics of ΔMAH in the presence of excess NAD^+^. *F*, steady-state kinetics of ΔMAH in the presence of excess 7α-OHC. HSD3B7, 3β-hydroxy-Δ^5^-C_27_-steroid oxidoreductase; 7α-OHC, 7α-hydroxycholesterol.

Membrane prediction servers identify a putative C-terminal membrane-associated helix (MAH) in HSD3B7 (Fig. 5*A*) (32). A homology model of HSD3B7 using AlphaFold3 (33) and Orientations of Proteins in Membranes (OPM) (34) database predict that this amphipathic helix (residues 289–313) is embedded in the membrane at a depth of 9.8 ± 1.2 Å. This MAH is uniquely positioned for either extracting hydrophobic ligands from the membrane of interest, for localization, or for both purposes. To determine if this helix plays a role in the mechanism of HSD3B7, we engineered HSD3B7 constructs using a combination of solubilizing mutations and an MAH deletion mutant to create two modified protein constructs. Previous attempts to purify an MAH deletion construct without stabilizing mutations were unsuccessful, resulting in insoluble protein. To increase the stability of these constructs, multiple sequence alignments of homologous proteins were used to determine a consensus sequence for HSD3B7 using Consensus Finder (35). Recommended mutations were determined by evaluating the frequency of each residue at a given position and predicting stabilization above a certain threshold of free energy of unfolding. Mutations within the predicted active site were not introduced, which resulted in 16 total suggested mutations distal to the predicted active site (Fig. S1*A*). The resulting consensus construct, cHSD3B7, was further modified with a flexible GGGS linker, removing residues 289 to 313 (Fig. S1*B*). This consensus MAH deletion construct, cHSD3B7ΔMAH, is no longer predicted to contain a C-terminal MAH (Fig. 5*B*) and could be purified to homogeneity in the absence of detergent from insect cells and eluted as a single peak from gel filtration (Fig. S1, *C* and *D*). cHSD3B7ΔMAH exists as a monomer according to light scattering, mass photometry, with a predicted molecular weight of 38 ± 8 kDa, with a calculated molecular weight of cHSD3B7ΔMAH of 42.6 kDa (Fig. 5*C*). This monomer state is comparable to full-length WT and cHSD3B7, which have molecular weights of 113 ± 21 kDa and 109 ± 25 kDa, respectively, when associated with *n*-dodecyl-d-maltopyranoside (DDM) micelles. The predicted molecular weight of a DDM micelle with a monomer of HSD3B7 is 113 kDa, which aligns well to the experimental values if HSD3B7 is associated with a micelle (Fig. S1*E*) (36).

To further validate this model, native-PAGE was utilized to verify oligomerization state of both HSD3B7 and cHSD3B7. Both HSD3B7 constructs migrate in a single band, suggesting a single oligomerization state (Fig. S1*F*). In contrast, HSD3B1 and HSD3B2 constructs result in multiple bands during native-PAGE, likely corresponding to monomer, dimer, and tetramer formation. To verify these results, we used chemical crosslinking to determine if we could trap these oligomers in solution. Both HSD3B1 and HSD3B2 show signs of dimer formation with the appearance of a band around 90 kD, whereas HSD3B7 in the presence of crosslinker does not result in higher order bands (Fig. S1*G*).

cHSD3B7ΔMAH appears to be thermostable with a melting temperature of 46.3 ± 0.2 °C and remains thermostable in the presence of substrates NAD^+^ (47.5 ± 0.0 °C) and 7α-OHC (47.4 ± 0.2 °C) (Fig. 5*D*). Compared with WT HSD3B7, this construct maintains a comparable *K*_*m*_ for 7α-OHC of 15.6 μM [9.5, 25.7] and only an approximately twofold higher *K*_*m*_ for NAD^+^ (101.3 μM [77.4, 133]) (Fig. 5, *E* and *F*). In contrast, there are major defects in catalysis, with a *k*_cat_ and catalytic efficiency that is 30-fold decreased from WT HSD3B7 (Table 3). By contrast, cHSD3B7 shows a slight increase in catalytic efficiency in comparison to WT HSD3B7 (Fig. S1, *H* and *I*, Table S1). Together, these data suggest that the HSD3B7 membrane helix is essential for catalysis and that solubilizing mutations can be tolerated.

**Table 3:** Kinetic constants for cHSD3B7-ΔMAH substrates

Substrate	*K*_*m*_ (μM)	*V*_max_ (nmol/min/mg)	*k*_cat_ (min^−1^)	*k*_cat_/*K*_*m*_ (M^−1^ s^−1^) × 10^4^
7α-OHC	15.6 (9.5, 25.7)	30.4 (26.2, 35.7)	0.15 (0.13, 0.17)	0.0160 ± 0.053
NAD^+^	101.3 (77.39, 133.0)	8.8 (8.0, 9.7)	0.044 (0.040, 0.048)	0.00072 ± 0.00025

### AlphaFold predictions suggest a membrane helix forms a tunnel for the oxysterol substrate

**Figure 6:**
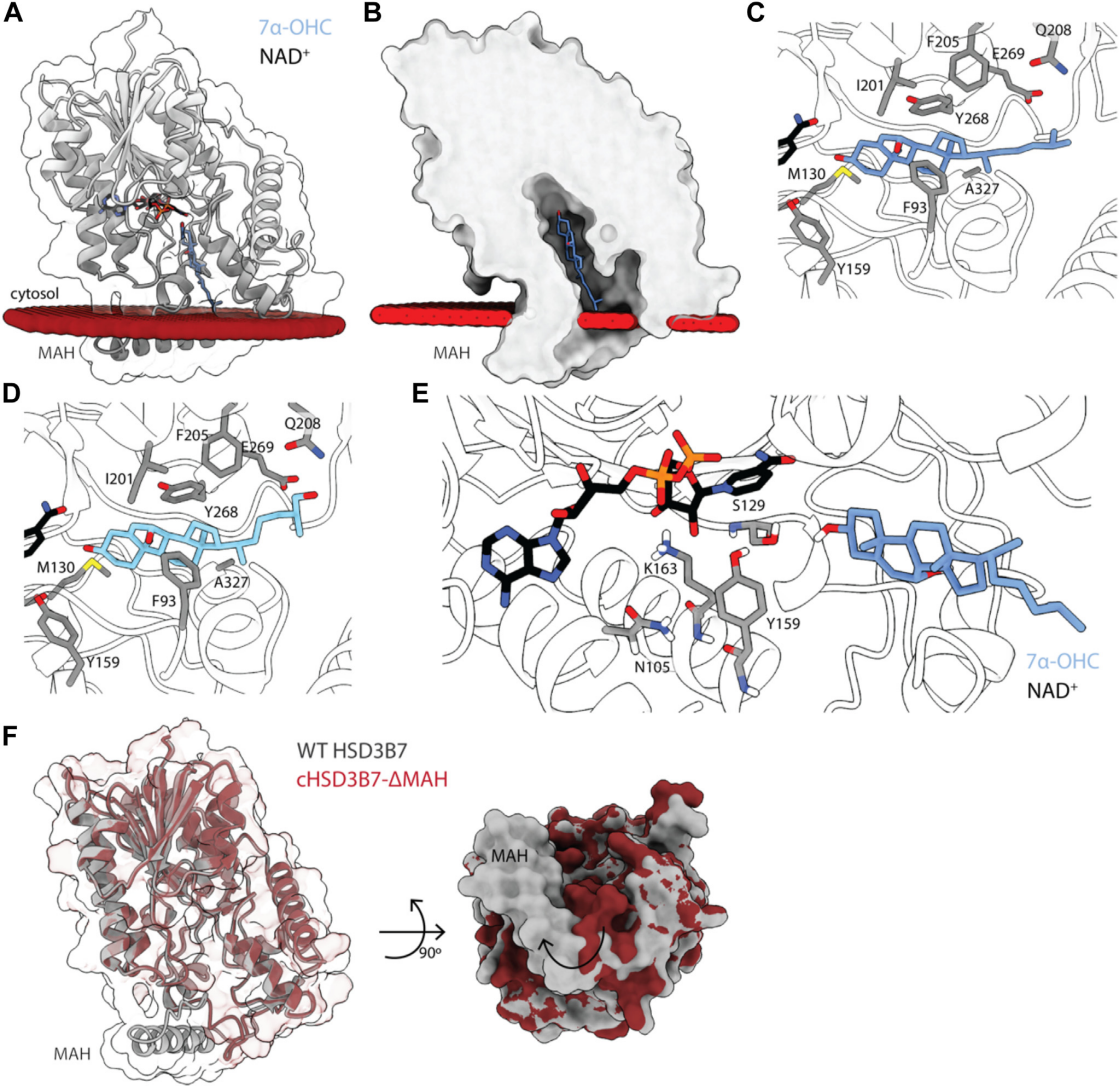
**Alphafold modeling and molecular docking of HSD3B7.***A*, WT HSD3B7 model with docking of substrates NAD^+^ and 7α-OHC. OPM membrane prediction is show in *red*, with HSD3B7 on the cytosolic side of the membrane. *B*, Surface rendering of WT HSD3B7 model depicting oxysterol pocket, with 7α-OHC docked within the pocket. *C*, docking pose of 7α-OHC within a predicted binding pocket with key interactions labeled. *D*, docking pose of 7α,27-diHC within a predicted binding pocket with key interactions labeled. *E*, Y-S-N-K catalytic tetrad of HSD3B7 in docking model of WT HSD3B7 with relative positioning to NAD^+^ and 7α-OHC. *F*, overlay of WT HSD3B7 and cHSD3B7-ΔMAH models. Rotated models show differences in access to the oxysterol binding pocket.HSD3B7, 3β-hydroxy-Δ^5^-C_27_-steroid oxidoreductase; 7α-OHC, 7α-hydroxycholesterol.

AlphaFold3 was utilized to predict models of the three constructs, WT HSD3B7, cHSD3B7, and cHSD3B7-MAH (Fig. S2, *A*–*C*) (33). Solubilizing mutations present in the cHSD3B7 construct do not appear to have any effect on overall HSD3B7 architecture with an overall RMSD of 0.167 Å, agreeing well with our kinetic data (Fig. S2*D*). Substrates, NAD^+^ and 7α-oxysterols, were docked into the predicted WT structure using AlphaFold3 and SwissDock, respectively (33, 37) (Fig. 6*A*). The membrane surface generated by the OPM prediction server is displayed, with the MAH embedded into it. Unsurprisingly, NAD^+^ is docked into the conserved Rossmann fold domain and is predicted to form key interactions with conserved NAD(H) binding residues (Fig. S2*E*). Top orientations for oxysterol substrates were selected, and conformations that fell within the conserved NAD^+^ pocket were excluded. These docking poses resulted in binding energies within the range of −8.7 to −8.1 kcal/mol (Table S2).

The WT HSD3B7 predicted structure rendered as a surface view reveals a tunnel that likely facilitates oxysterol extraction from the membrane (Fig. 6*B*). The membrane surface is perpendicular to the oxysterol pocket, suggesting that the location of the MAH assists in forming the base of this tunnel. Upon docking of the oxysterol substrates of HSD3B7, we observe binding posies of these oxysterols within the pocket that were consistent (Fig. 6, *C* and *D*). All oxysterols appear to take advantage of similar hydrophobic and hydrogen bonding interactions (Fig. S2, *F* and *G*). Specifically, all oxysterol substrates are predicted to have hydrophobic interactions with F93, F205, and I201. The similarities between the positioning of the 7α hydroxyl group containing oxysterols within the tunnel is consistent with the similar *K*_*m*_ values that we determine for these substrates. The slight preference seen by HSD3B7 for 7α,27-diHC could be explained through productive hydrogen bonding between the C_27_-hydroxyl group and Q208 (Fig. 6*D*). In addition, the 3β hydroxy groups of the oxysterols are in close proximity to the highly conserved Y-N-S-K catalytic tetrad present in most SDR family enzymes (Fig. 6*E*).

The substrate pocket seen in the model of WT HSD3B7 is not observable in the AlphaFold predictions of cHSD3B7-MAH, which could help explain the role of this tunnel in the correct positioning of the substrate to maintain catalytic efficiency (Fig. 6*F*). The prediction in this region of cHSD3B7-MAH is less confident, likely beacause of the inherent flexibility built into the linker (Fig. S2*C*). Despite similar *K*_*m*_ values of oxysterol substrates for both WT HSD3B7 and cHSD3B7-MAH (Fig. 5*E*), the roughly 30-fold decrease in catalytic efficiency exhibited by the deletion construct suggests that the MAH domain plays a crucial role in positioning the substrate for catalysis.

### Predicted oxysterol binding pocket residues impact HSD3B7 activity

**Figure 7:**
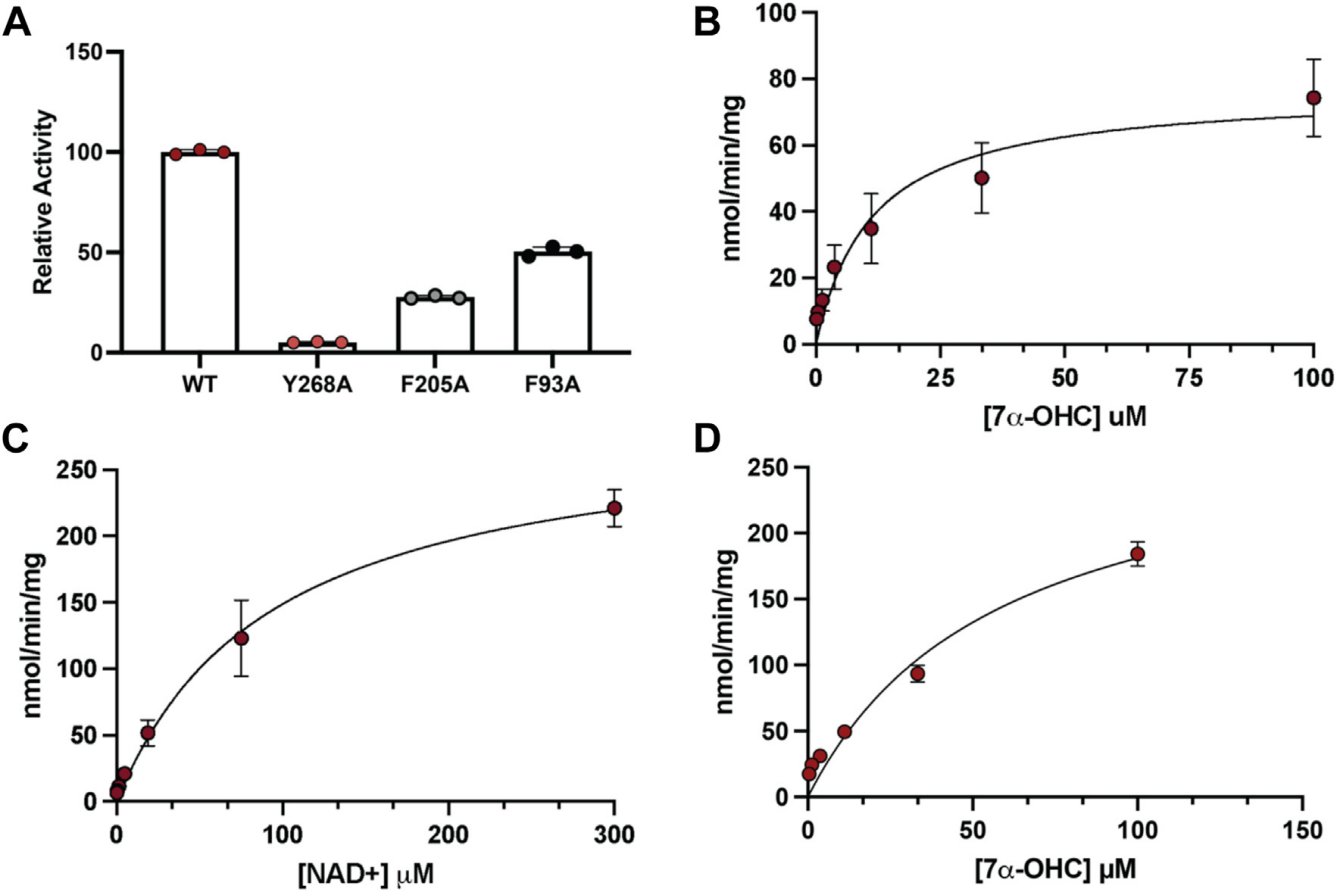
**HSD3B7 mutant activity and steady-state kinetics.***A*, relative activity of HSD3B7 predicted oxysterol binding pocket mutants. *B*, steady-state kinetics of F205A mutant in the presence of excess NAD^+^. *C*, steady-state kinetics of F205A mutant in the presence of excess 7α-OHC. *D*, F93A mutant activity in the presence of excess NAD^+^. HSD3B7, 3β-hydroxy-Δ^5^-C_27_-steroid oxidoreductase; 7α-OHC, 7α-hydroxycholesterol.

We evaluated docking of the 7α-OHC substrate by conducting site-directed mutagenesis of residues identified in our docking experiments, including Y268A, F205A, and F93A (Fig. 6*D*). All three mutants were purified to homogeneity and have decreases in relative activity in comparison to WT HSD3B7 (Fig. 7*A*). Steady-state kinetics could not be determined for Y268A since enzyme activity was barely detectable. Steady-state kinetics for F205A gave similar *K*_*m*_ values for both cosubstrates, 7α-OHC and NAD^+^, to that of WT enzyme (Fig. 7, *C* and *D*, Table 4). In contrast, there were significant defects in *k*_cat_, with over 5- to 10-fold decrease in catalytic efficiency for F205A suggesting that the reaction trajectory may have been affected (Table 4). For the F93A mutant, we calculated an apparent *K*_m_ for 7α-OHC of about 58 μM (∼3-fold higher than WT because of its limited solubility and also meant the kinetic studies could not be completed since the enzyme could not be saturated with NAD^+^) (Fig. 7*D*, Table 5). Together, these data are consistent with the docking of 7α-OHC in HSD3B7.

**Table 4:** Kinetic constants for F205A

Substrate	*K*_*m*_ (μM)	*V*_max_ (nmol/min/mg)	*k*_cat_ (min^−1^)	*k*_cat_/*K*_*m*_ (M^−1^ s^−1^) × 10^4^
7α-OHC	11.2 (5.2, 23.9)	76.6 (62.5, 96.0)	1.5 (1.3, 1.9)	0.22 ± 0.07
NAD^+^	93.8 (67.0, 133.3)	288.2 (255.0, 330.6)	5.8 (5.1, 6.6)	0.10 ± 0.04

**Table 5:** Kinetic constants for F93A

Substrate	*K*_*m*_ (μM)	*V*_max_ (nmol/min/mg)	*k*_cat_ (min^−1^)	*k*_cat_/*K*_*m*_ (M^−1^ s^−1^) × 10^4^
7α-OHC	58.2 (33.4, 112.4)	286.5 (224.2, 405.0)	14.3 (11.2, 20.3)	0.41 ± 0.20

## Discussion

The recently reported role of HSD3B7 in ccRCC and the established supplementation of cholic acid treatment for HSD3B7 deficiencies suggests that the activity of HSD3B7 could be manipulated for therapeutic purposes. However, a thorough kinetic analysis of HSD3B7 was lacking, which is vital for informed development of HSD3B7 inhibitors. To this end, we characterized the substrate specificity of HSD3B7 through a series of steady-state kinetic measurements. These assays demonstrate that HSD3B7 can catalyze reduction and by inference isomerization reactions on diverse 7α hydroxy oxysterols and depend on membrane association through a putative MAH.

Evaluation of steady-state kinetic parameters revealed that HSD3B7 can catalyze the dehydrogenation and isomerization of multiple different 7α-hydroxy substrates with similar catalytic efficiencies. In alignment with the literature, the determined kinetic values fall in the low micromolar range for *K*_*m*_ (24, 25, 26). Interestingly, pig HSD3B7 was reported to have 8- and 45-fold lower *k*_*cat*_ values toward 7-DOCA and 7α,25-diHC, which is not seen for the human enzyme (26). This could suggest evolutionary differences in substrate specificity and availability. The ability of human HSD3B7 to catalyze these reactions is expected given that this broad substrate specificity is crucial for the synthesis of primary bile acids. As a versatile enzyme, HSD3B7 is able to accommodate oxysterols made from both the classic and acidic bile acid pathways, with the requirement for a 7α hydroxy group. This broad substrate specificity is similar to other 3β-HSD enzymes, HSD3B1 and HSD3B2, which catalyze similar reactions on C_19_ and C_21_ containing sterols, whose substrates differ in modifications of the steroid ring and at the 17 position (9, 30, 38). In addition, we aimed to understand the oligomerization state of HSD3B7 and compared our methods to other 3β-HSD enzyme family members, which highlighted some differences. Using multiple methods, including native-PAGE and chemical crosslinking, we have evidence that HSD3B7 is a monomer in solution, whereas HSD3B1 and HSD3B2 appear to form higher order oligomers. This will need to be further verified using structural methods.

Similarly to HSD3B1, HSD3B7 shows low micromolar affinity toward NADH, which is thought to be essential for the activation of the isomerization reaction (39). Considering their sequence and structural homology, it is highly likely that these 3β-HSD enzymes share conserved catalytic mechanisms that would involve the Y-S-N-K tetrad for dehydrogenation and a separate Y for the isomerization reaction. Our docking experiments reveal the 3β hydroxy is in close proximity to the catalytic tetrad. In the absence of a structure, homology models were utilized to make predictions about substrate binding modes and specificity. Based on these results, we can speculate that the binding pocket is able to tolerate additional oxidation of oxysterol substrates by taking advantage of hydrogen bonding with polar residues within the binding pocket. To definitively answer these questions, future structural studies will be necessary. In addition, we were able to verify the role of critical residues within the predicted oxysterol binding pocket, which were found to have decreased activity in comparison to WT enzyme supporting the docking model.

In addition to relying on a versatile oxysterol binding pocket, HSD3B7 likely catalyzes a sequential ordered substrate binding mechanism. Consistent with other members of the 3β-HSD and SDR enzyme family, HSD3B7 binds NAD^+^ first and followed by sterol substrate (27, 39, 40). It is also likely that the mechanism is an ordered bi–bi since the free enzyme can bind the product NADH. Recently, related NAD(P)-dependent steroid dehydrogenase-like, NSDHL, was found to undergo a conformational change upon NAD^+^ binding that allowed for substrate binding (41). We can speculate that a similar event occurs in the catalytic cycle of HSD3B7, where NAD^+^ binding induces a conformational change that allows for oxysterol binding.

HSD3B7 also contains an MAH that regulates enzyme function. Upon deletion of this helix, HSD3B7 showed significant impairment in catalytic activity and modestly higher *K*_*m*_ values toward NAD^+^. The homology model of WT HSD3B7 shows a substrate tunnel that forms near the predicted membrane interface. In the absence of this helix in the cHSD3B7ΔMAH model, there is likely more flexibility within the pocket area, potentially leading to less stable tunnel formation. There are a few possibilities that could explain why this helix is essential for catalysis. The observed decrease in *k*_cat_ suggests that in addition to its role of orienting and bringing hydrophobic substrates in proximity to the catalytic tetrad, it might also be involved in a conformational change necessary for product release. This is in contrast to paralog HSD3B1, which showed no difference in substrate affinity or catalytic efficiency upon deletion of the corresponding MAH (42, 43). In addition, HSD3B7 required additional stabilizing mutations to maintain solubility, further suggesting differences in nonconserved residues within the 3β-HSD family to play a role in overall protein stability.

As a recently discovered therapeutic target, small molecule inhibition of HSD3B7 is a promising opportunity for drug development. Understanding the substrate preference of HSD3B7 provides an advantage for the development of highly specific and potent inhibitors. Specifically, a sequential ordered bi–bi mechanism would allow for future drugs to be designed to inhibit individual complexes (*e.g.* E.NAD^+^; E.NADH) in the mechanism to allow for more specific and on-target inhibition. Future studies could focus on structure determination of HSD3B7 and further optimization of current known inhibitors.

## Experimental procedures

### HSD3B7 construct expression and purification

WT HSD3B7 and cHSD3B7 was cloned into a pFastBac vector with an N-terminal 6x histidine tag and a tobacco etch virus cleavage site. cHSD3B7 gene was purchased from GenScript. Recombinant HSD3B7 was produced using an Sf9 (*S*. *frugiperda*) expression system (ThermoFisher, catalog no.: 12659017). High density (2 × 10^6^ cells ∗ mL^−1^) suspension cultures of Sf9 cells were infected in Fernbach shake flasks at 27 °C. Cells were harvested after 48 h and resuspended in lysis buffer (20 mM potassium phosphate [pH 7.4], 20% glycerol) that was supplemented with DNAse and cOmplete EDTA-free Protease Inhibitor cocktail (Roche). Cells were lysed with sonication and clarified at 30,000*g*. The membrane fraction was isolated from the supernatant *via* ultracentrifugation at 110,000*g*. Membrane pellets were resuspended in 20 mM potassium phosphate (pH 7.4), 20% glycerol, and solubilized with addition of 1% DDM (Anatrace). Membranes were lightly rocked at 4 °C for 2 to 6 h. The insoluble fraction was pelleted at 50,000*g*, and the supernatant was passed over Ni–NTA resin (Thermo Scientific), which was washed with ∼20 CV of wash buffer (20 mM potassium phosphate [pH 7.4], 10% glycerol, and 0.04% DDM) and eluted with elution buffer (20 mM potassium phosphate [pH 7.4], 10% glycerol, 0.04% DDM, and 200 mM imidazole). Eluate was concentrated using a 50 KDa molecular weight cutoff (Amicon Ultra; Millipore) and loaded onto a S200prep gel-filtration column (GE Healthcare) in a buffer containing 20 mM potassium phosphate (pH 7.4), 10% glycerol, and 0.04% DDM. Protein was snap-frozen in liquid nitrogen and stored at −80 °C.

Site-directed mutagenesis was utilized to create the cHSD3B7-ΔMAH deletion construct. Primers flanked the membrane helix at residues 288 and 314 and added a GGGS linker (Forward: 5′-GGTGGCGGATCTCTCTATGCACCGTTGC-3′, Reverse: 5′-TGTAGGATCTAGGCCC-3′). This gene was then cloned into a pFastBac vector with an N-terminal 6x histidine tag and a tobacco etch virus cleavage site. Recombinant cHSD3B7-ΔMAH was produced using an Sf9 expression system (ThermoFisher, catalog no.: 12659017). High density (2 × 10^6^ cells ∗ mL^−1^) suspension cultures of Sf9 cells were infected at a multiplicity of infection of 1 in Fernbach shake flasks at 27 °C. Cells were harvested after 48 h and resuspended in lysis buffer (20 mM Hepes [pH 7.5], 150 mM NaCl, 2 mM 2-beta-mercaptoethanol [βME], 10 mM imidazole [pH 7.5], and 20% glycerol) that was supplemented with DNAse and complete EDTA-free Protease Inhibitor cocktail (Roche). Cells were lysed with sonication and clarified at 50,000G, and supernatant was passed over Ni–NTA resin, which was washed with ∼20 CV of wash buffer (20 mM Hepes [pH 7.5], 150 mM NaCl, 2 mM βME, 10 mM imidazole [pH 7.5], and 10% glycerol) and eluted with elution buffer (20 mM Hepes [pH 7.5], 150 mM NaCl, 2 mM βME, 250 mM imidazole [pH 7.5], and 10% glycerol). Eluate was concentrated using a 30 kDa molecular weight cutoff and loaded onto a S200prep gel-filtration column (GE Healthcare) in a buffer containing 20 mM Hepes (pH 7.5), 150 mM NaCl, 1 mM Tris(2-carboxyethyl)phosphine, and 10% glycerol. Protein was snap-frozen in liquid nitrogen and stored at −80 °C.

### Consensus mutations of HSD3B7 protein sequence

Consensus Finder (University of Minnesota) was utilized to predict stabilizing mutations of HSD3B7. This online server predicted a consensus sequence and recommended a total of 16 substitutions that would be likely to increase stability of HSD3B7. Recommended mutations that were close to active site were removed from consideration.

### WT HSD3B7 kinetic assay

Assays were carried out at 25 °C in a reaction buffer (RB) containing 20 mM potassium phosphate (pH 7.4), 10% glycerol, 0.04% DDM with a final well volume of 12.5 μl in triplicate. Reaction conditions contained 20 nM HSD3B7, 0.8 to 200 M NAD^+^ (EMD Millipore), and 0.04 to 100 μM oxysterols, 7α-OHC (Focus Biomolecules), 7-DOCA, 7α,25-dihydroxycholesterol (MedChemExpress), and 7α,27-dihydroxycholesterol (Avanti Lipids). NAD^+^ or oxysterol cosubstrates were held at 5x excess of *K*_*m*_, whereas other substrates were varied. Reactions were initiated by the addition of NAD^+^ and were stopped after 10 min by adding 5 μl of NAD(P)H Glo Detection Reagent (Promega). Values were background subtracted and fit to the Michaelis–Menten equation using Graphpad Prism 10.0.

### Microscale thermophoresis

All MST assays were carried out using a Nanotemper MST Monolith NT.115^pico^ with monolith premium capillaries. HSD3B7 K163R was labeled using the nanotemper second-generation RED-Tris-NTA His-Tag Labeling Kit according to the manufacturer's specifications. To check 7α-OHC binding, 20 nM HSD3B7 was mixed 1:1 with either matched buffer or 300 μM 7α-OHC and placed into capillaries in quadruplicates, resulting in final concentrations of 10 nM HSDB7 and 150 μM 7α-OHC. To check the binding of NAD^+^ and 7α-OHC, 20 nM HSD3B7 was preincubated with 2 mM NAD^+^ and mixed 1:1 with matched buffer or 300 μM 7α-OHC in quadruplicate, resulting in final concentrations of 10 nM HSD3B7 with 1 mM NAD^+^ and 150 μM 7α-OHC. Fluorescence was normalized using MO Control Software. Data were plotted and analyzed in Graphpad Prism 10.0. An unpaired *t* test was utilized to analyze differences in relative fluorescence between conditions.

### NADH *K*_*d*_ determination

A 5 μl reaction was excited at 285 nm, and emission scans were collected over 300–500 nm in duplicate. Reaction conditions were NADH (10 μM–150 nM in RB containing 20 mM potassium phosphate [pH 7.4], 10% glycerol, and 0.04% DDM) with or without HSD3B7 (1.8 μM in RB). The difference in fluorescence at 460 nm was calculated for each condition by subtracting the fluorescence signal in the absence of NADH. *F*_max_ is the maximum change in fluorescence at saturation. *F/F*_max_ was plotted as a function of NADH concentration and fit to the Morrison equation using Graphpad Prism 10.0.

(1a) $$\Delta F/\Delta F_{\max}=\left[\text{EC}\right]/\left[E\right]$$

(1b) $$\left[\text{EC}\right]=\left\{K_{d}+\left[E\right]+\left[C\right]–\;{\left[{\left(K_{d}+\left[E\right]+\left[C\right]\right)}^{2}\;–\;4\left[E\right]\left[C\right]\right]}^{1/2}\right\}/2$$

### Mass photometry

Mass photometry was performed using a Refeyn TwoMP. HSD3B7 samples were diluted in respective RB to a concentration of 100 nM. The instrument was calibrated using the manufacturer's suggestions, and sample was diluted in drop to a final concentration of 20 nM. About 60 s videos were collected using Refeyn AcquireMP, and the mass was fit using analysis tools in Refeyn DiscoverMP.

### Differential scanning fluorimetry

About 0.5 mg/ml cHSD3B7-MAH was added to either 50 μM 7α-OHC or 800 μM NAD^+^ with 5X SYPRO orange protein gel stain (Thermo Fisher Scientific; catalog no.: S6650) and added to a clear 384-well plate and covered with an optically clear cover. Plates were analyzed on a ViiA7 QPCR with a starting temperature of 20 and ramp rate of 0.05. Output was analyzed with TSA-CRAFT, and *T*_*m*_ values were determined and plotted with Graphpad Prism 10.

### cHSD3B7MAH kinetic assay

Assays were carried out at 25 °C in an RB containing 25 mM Hepes (pH 7.5), 10% glycerol, and 150 mM NaCl (RB2) in a final well volume of 12.5 μl in triplicate. Reaction conditions contained 200 nM HSD3B7, 0.8 to 500 μM NAD^+^, and 0.04 to 100 μM of 7α-OHC. NAD^+^ or oxysterol cosubstrates were held at 5x excess of *K*_*m*_, whereas the other substrate was varied. Reactions were initiated by the addition of NAD^+^ and were stopped after 10 min by adding 5 μl of NAD(P)H Glo Detection Reagent. Values were background subtracted and fit to the Michaelis–Menten equation using Graphpad Prism 10.0.

### cHSD3B7 kinetic assay

Assays were carried out at 25° in an RB in a final well volume of 12.5 μl in triplicate. Reaction conditions contained 20 nM HSD3B7, 0.8 to 500 μM NAD^+^, and 0.04 to 100 μM of 7α-OHC. NAD^+^ or oxysterol cosubstrates were held at 5x excess of *K*_*m*_, whereas other substrates were varied. Reactions were initiated by the addition of NAD^+^ and were stopped after 10 min by adding 5 μl of NAD(P)H Glo Detection Reagent. Values were background subtracted and fit to the Michaelis–Menten equation using Graphpad Prism 10.0.

### AlphaFold

AlphaFold3 was utilized to predict structures of three constructs, WT HSD3B7, cHSD3B7, and cHSD3B7-ΔMAH. The top ranked structure was used in all visualization and docking experiments.

### Docking

SwissDock online server was utilized to run AutoDock Vina on HSD3B7 and oxysterol substrates. The WT HSD3B7 AlphaFold model was used as receptor, and oxysterol ligand files were prepared using PyMOL by Schrodinger. The docking grid was a box size of 19 Å^3^ and manually placed in the active site of HSD3B7. The docking parameters were set to low exhaustivity and random initial conditions of 1. Resulting poses that showed oxysterol in the binding pocket with correct orientation for positioning of 3β hydroxyl were curated and visualized using UCSF ChimeraX.

### Relative activity assays

About 50 nM of each enzyme, WT, Y268A, F205A, and F93A were incubated with 80 μM 7α-OHC in RB, and reactions were started by the addition of 300 μM NAD^+^ in a final volume of 10 μl. Reactions were run for 10 min and stopped with the addition of 5 μl of NAD(P)H-Glo reagent as previously described. Values were normalized to WT protein and plotted using Graphpad Prism 10.0.

## Supporting information

This article contains supporting information.

## Conflict of interest

The authors declare that they have no conflicts of interest with the contents of this article except for T.M.P. who is the cofounder of Penzymes and is a consultant for Sage Therapeutics and Propella, Inc.
