# Supplementary material for: Substrate specificity and kinetic mechanism of 3β-hydroxy-Δ5-C27-steroid oxidoreductase
Source: J Biol Chem. 2024 Nov 4;300(12):107945. doi: 10.1016/j.jbc.2024.107945 (PMC11648244; doi:10.1016/j.jbc.2024.107945)
Supplement: Supporting information [file mmc1.pdf]

Supplemental Information

Supplemental Table 1 Kinetic Constants for cHSD3B7

	K_m (μM)	V_{max} (nmol/min/mg)	k_{cat} (min^{-1})	k_{cat} / K_m (M^{-1} s^{-1}) x 10^4
7α-OHC	9.0 [5.2, 15.6]	757.8 [650.5, 887.3]	37.9 [32.5, 44.3]	7.0 ± 2.0
NAD^+	10.8 [8.4, 13.7]	885.3 [839.1, 933.1]	44.3 [42.0, 46.7]	6.8 ± 1.5

Supplemental Table 2 Binding energies of oxysterol docking into WT HSD3B7

Substrate	SwissParam Score
7α-OHC	-8.3
7-DOCA	-8.4
7α,25-diHC	-8.7
7α,27-diHC	-8.1

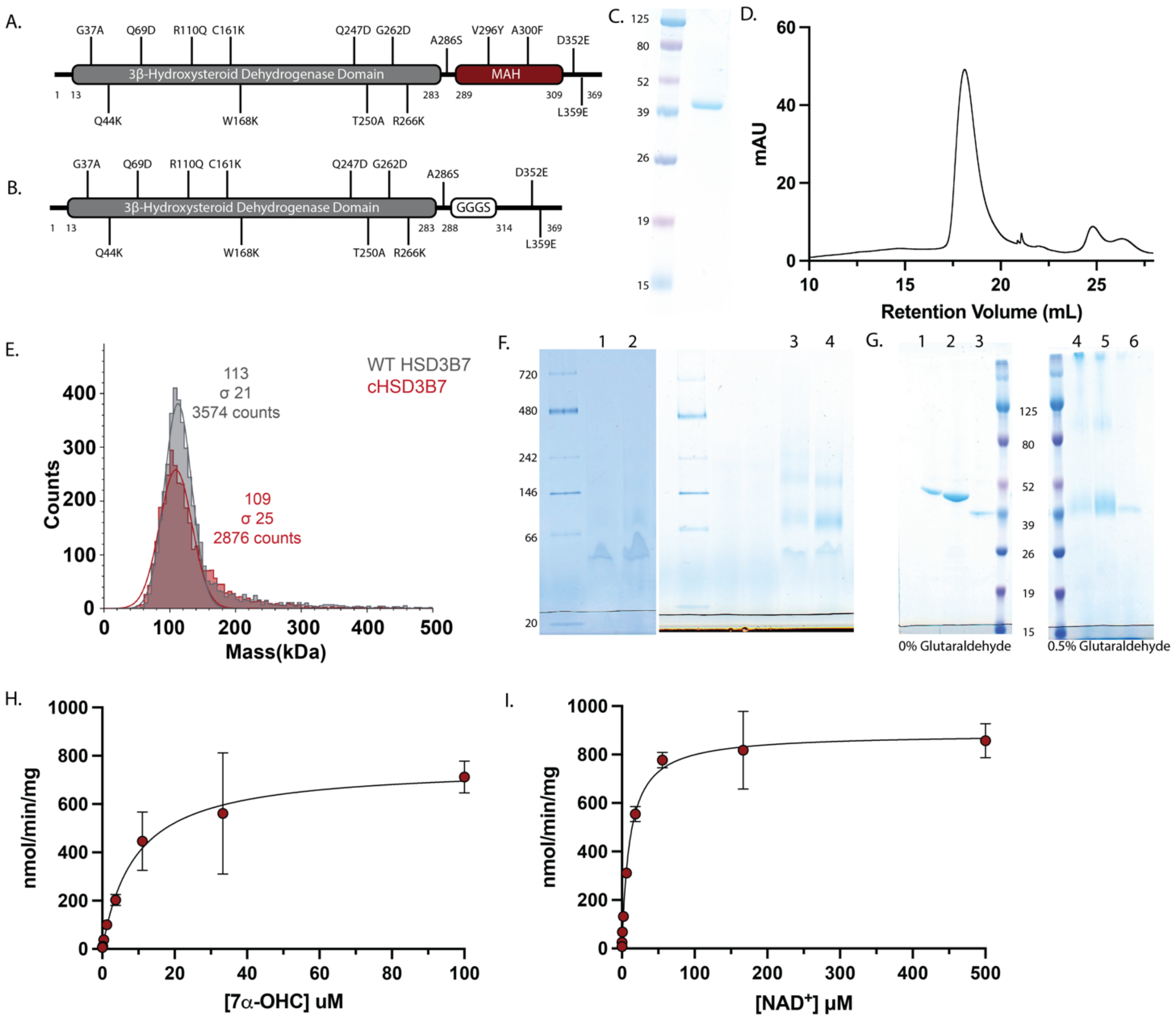

Supplemental Figure 1 Consensus constructs of HSD3B7 and substrate kinetics(A) Consensus mutations present in cHSD3B7 construct. (B) Consensus mutations present in the cHSD3B7-ΔMAH construct. (C) SDS-PAGE gel of ΔMAH construct. (D) S200 gel filtration chromatogram of ΔMAH purification. (E) Mass photometry of WT HSD3B7 (gray) and cHSD3B7 (red) with predicted molecular weights of 113 and 109 kDa respectively. (F) Native-PAGE of WT HSD3B7 (lane 1), cHSD3B7 (lane 2), HSD3B1 (lane 3) and HSD3B2 (lane 4). (G) Crosslinking assay of HSD constructs in the presence (lanes 4-6) and absence (lanes 1-3) of 0.5% glutaraldehyde. Lane 1-3 depicts HSD3B1, HSD3B2 and HSD3B7 respectively. Lanes 4-6 depicts HSD3B1, HSD3B2 and HSD3B7 respectively. (H) Steady state kinetics of cHSD3B7 in excess of NAD^+. (I) Steady state kinetics of cHSD3B7 in the presence of excess 7α-OHC

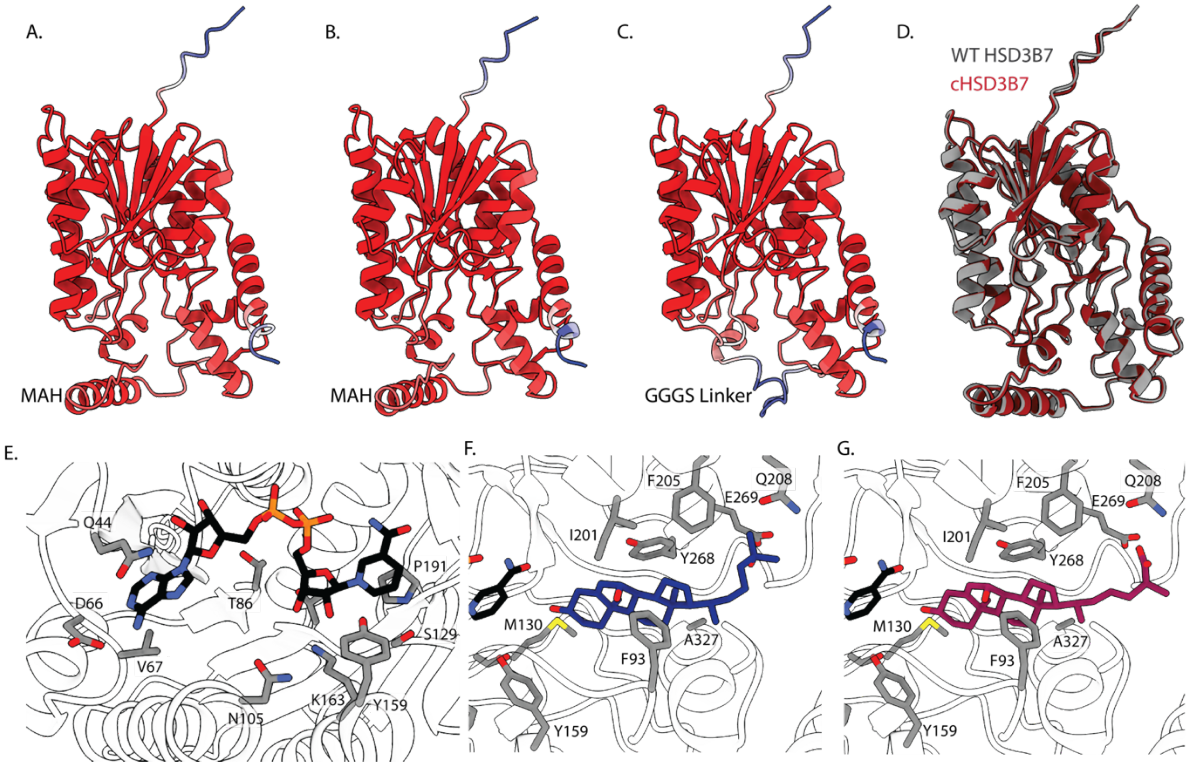

Supplemental Figure 2 Alphafold model prediction and substrate docking(A) AlphaFold confidence in prediction of WT HSD3B7 model. Red is high confidence; blue is low confidence. (B) AlphaFold confidence in prediction of cHSD3B7 model. (C) AlphaFold confidence in prediction of cHSD3B7-ΔMAH model. (D) Overlay of WT HSD3B7 and cHSD3B7 alphafold predictions. (E) NAD^+ docking orientation in WT HSD3B7 with important interactions labeled. (F) Docking pose of 7α,25-diHC within predicted binding pocket with key interactions labeled. (G) Docking pose of 7-DOCA within the predicted binding pocket with key interactions labeled.
